# Five-year Follow-up of Transobturator Sling: 152 Cases with the Same Surgeon

**DOI:** 10.1055/s-0038-1670712

**Published:** 2018-10

**Authors:** Mucio Barata Diniz, Luisa Campos Barata Diniz, Gustavo Francisco Lopes da Silva, Agnaldo Lopes da Silva Filho, Zilma Silveira Nogueira Reis, Marilene Vale de Castro Monteiro

**Affiliations:** 1 Departament of Gynecology and Obstetrics, Universidade Federal de Minas Gerais, Belo Horizonte, MG, Brazil; 2 Hospital Vila da Serra, Belo Horizonte, MG, Brazil

**Keywords:** stress urinary incontinence, suburethral support, transobturator sling, incontinência urinária de esforço, suporte suburetral, *sling* transobturatório

## Abstract

**Objective** To evaluate the long-term subjective cure rate of the transobturator sling, including an analysis of the risk factors and of the impact of increased surgical experience on the results.

**Methods** A retrospective cohort study of women who underwent transobturator sling surgery from 2005 to 2011 was conducted. Patients were evaluated by a telephone survey using the International Consultation on Incontinence Questionnaire-Short Form (ICIQ-SF) and by subjective questions regarding satisfaction. An ICIQ-SF score of 0 was considered a cure. The crude and adjusted odds ratios and 95% confidence intervals were estimated in univariate and multivariate logistic regression models to identify risk factors for surgical failure. Differences with *p* < 0.05 were considered significant.

**Results** In total, 152 (70.6%) patients answered the questionnaire. The median follow-up period was 87 months. The urodynamic diagnosis was stress urinary incontinence in 144 patients (94.7%), and mixed urinary incontinence in 8 (5.3%) patients. Complications occurred in 25 (16%) patients. The ICQ-SF results indicated that 99 (65.10%) patients could be considered cured (ICIQ-SF score = 0). Regarding the degree of satisfaction, 101 (66%) considered themselves cured, 43 (28%) considered themselves improved, 7 (4.6%) considered themselves unchanged, and one reported worsening of the incontinence. After the univariate and multivariate analyses, the primary risk factor for surgical failure was the presence of urgency (*p* < 0.001).

**Conclusion** The transobturator sling is effective, with a low rate of complications and a high long-term satisfaction rate. The risk factors for failure were the presence of urgency and patient age. The increased experience of the surgeon was not a factor that influenced the rate of complications.

## Abstract (pt)

**Resumo:**

**Objetivo** Avaliar a taxa de cura subjetiva do *sling* transobturatório no longo prazo, incluindo a análise dos fatores de risco e o impacto da experiência do cirurgião nos resultados.

**Métodos** Foi realizado um estudo de coorte retrospectivo em mulheres submetidas à cirurgia do *sling* transobturatório entre 2005 e 2011. As pacientes foram avaliadas por meio de uma entrevista aplicada por telefone utilizando o International Consultation on Incontinence Questionnaire-Short Form (“Questionário Internacional de Consulta sobre Incontinência – Forma Abreviada”, ICIQ-SF, na sigla em inglês) acrescido de questões sobre a satisfação das pacientes com a cirurgia. O critério de cura utilizado foi o ICIQ-SF = 0. A razão de chances bruta e ajustada e intervalos de confiança de 95% foram estimados por meio de modelos de regressão logística univariada e multivariada para identificar fatores de risco para falha cirúrgica. Diferenças com *p* < 0,05 foram consideradas significativas.

**Resultados** No total, 152 (70,6%) pacientes responderam. A média do período de acompanhamento foi de 87 meses. O diagnóstico urodinâmico foi incontinência urinária de esforço em 144 pacientes (94,7%) e incontinência urinária mista em 8 (5,3%). Complicações ocorreram em 25 (16%) pacientes. Os resultados do ICQ-SF indicaram que 99 (65,10%) pacientes estavam curadas (pontuação no ICIQ-SF = 0). Quanto ao grau de satisfação, 101 (66%) consideraram-se curadas, 43 (28%) melhoraram, em 7 (4,6%) não houve melhora, e uma relatou piora da incontinência. Após análise univariada e multivariada, o principal fator de risco para falha cirúrgica foi a presença de urgência (*p* < 0,001).

**Conclusão** O *sling* transobturatório é eficaz, tem baixo índice de complicações, e alto índice de satisfação no longo prazo. Os fatores de risco para falha foram a presença de urgência e a idade da paciente. O aumento da experiência do cirurgião não foi um fator que influenciou na taxa de complicações.

## Introduction

Urinary incontinence (UI) is a frequent condition in the female population, and, although it is considered benign, the symptoms impact women socially, physically, and psychologically.^1^ Surgical treatment should be offered to patients with stress urinary incontinence (SUI) or mixed urinary incontinence (MUI) who have not improved with the conservative treatment.^2^ Current evidence suggests that midurethral slings (MUS) have become the treatment of choice and are considered the gold standard.^3^ ^4^ Consistent long-term results indicate the effectiveness of tension-free vaginal tape (TVT) and of transobturator tape (TOT) for the MUS procedure.^5^ ^6^ The MUS is safe whether implemented via the retropubic or transobturator routes, and is effective in the short and medium terms. The TOT can be outside-in or inside-out, with no difference in cure rates between them. In the long term, subjective cure rates ranged from 43 to 92% in the transobturator route (TOR) group, and from 51 to 88% in the retropubic route (RPR) group.^1^ There is some evidence that it is also effective in the long term, but more long-term data are needed to clarify the uncertainties regarding the adverse effects and efficacy of the TOT.^7^

For women undergoing surgery to treat SUI, the long-term outcome probably means decades; however, there is little evidence-based research with adequate methodology that reports results beyond 5 years.^8^ It is important to establish the efficacy and long-term complications of urogynecological surgical treatments, especially for those procedures that use meshes. Collection and publication of data regarding the possible long-term complications related to the use of prosthetic materials is critical.^9^

The main objective of the present study was to evaluate the long-term (longer than 5 years) subjective cure rate for the transobturator sling in a group of patients operated on by the same surgeon. The secondary objectives were to evaluate whether increased surgical experience influenced the subjective cure rate and occurrence of complications, to verify the risk factors for failure, and to verify patient satisfaction with the technique.

## Methods

The present observational retrospective cohort study was approved by the Ethics in Research Committee of Universidade Federal de Minas Gerais (UFMG, in the Portuguese acronym), in the state of Minas Gerais, Brazil (under CAAE 22108413.30000.5149), and was conducted between July and December 2016. All patients who had surgical correction for UI by the author using the transobturator sling technique between 2002 and 2011 had their records reviewed. In all patients we used the unitape sling (Promedom, Rio de Janeiro, RJ, Brazil). All TOTs were outside in. Demographic data, clinical and urodynamic diagnoses, and intraoperative and postoperative data were collected. Two researchers, other than the surgeon, contacted each patient via telephone and invited them to participate in the study. After their acceptance, the informed consent was recorded. The patients then answered a telephonic survey with the questions of the International Consultation on Incontinence Questionnaire-Short Form (ICIQ-SF), as well as questions regarding their current degree of satisfaction with the surgery results. All of the nomenclature used followed the latest version of the terminology standardization of the International Continence Society.^10^

Patients with an urodynamic diagnosis of UI or MUI with predominant SUI who had undergone surgical correction using the synthetic transobturator sling technique at least 5 years earlier by a single surgeon (Diniz, MB) were qualified to participate. Patients with previous surgeries for UI and those with MUI or concomitant prolapse surgery were included. All patients provided detailed histories, and the hospital data included physical examination findings, with prolapse staging, urinalysis, and complete urodynamics.

Patients who had an ICIQ-SF score of 0 were considered cured. For the analysis of the patients' satisfaction with the surgery, we analyzed the answers to “I feel cured, improved, unchanged, or worse after surgery.” The effect of increased surgical experience was evaluated by comparing the means of subjective cure/failure rates per year and a graphical analysis of the number of surgeries per year and the complications and failures per year.

All variable categories were evaluated according to their absolute and relative frequencies. For the descriptive analysis of variables with a normal distribution, the results were expressed as mean ± standard deviation (SD). When the variable presented an asymmetric distribution, the results were expressed as the median and interval (minimum and maximum). The odds ratio (OR) was used to determine the prognostic values of the exposure factors (preoperative variables) to identify the risk factors for surgical failure (outcome).

Univariate and multivariate logistic regression models were employed to estimate the raw OR, and were then adjusted for cofactors with 95% confidence intervals (95%CIs). The multivariate analysis included all independent preoperative and postoperative variables from the univariate models, with individual importance in the univariate analysis. The set of variables that could best explain the occurrence of surgical failure was obtained by the enter method input, considering a *p* value of 0.20. The fit of the models and calibration, specifically the Hosmer-Lemeshow goodness-of-fit test and the adjusted coefficient of determination (adjusted R^2^), were performed based on the hypothesis that all coefficients were 0. In all statistical calculations, the level of significance was 0.05, and the 95%CI was 0.95. The statistical analysis was performed using the Statistical Package for the Social Sciences (SPSS Statistics for Windows, IBM Corp., Armonk, NY, US) software, version 21.0.

## Results

**Fig. 1:**
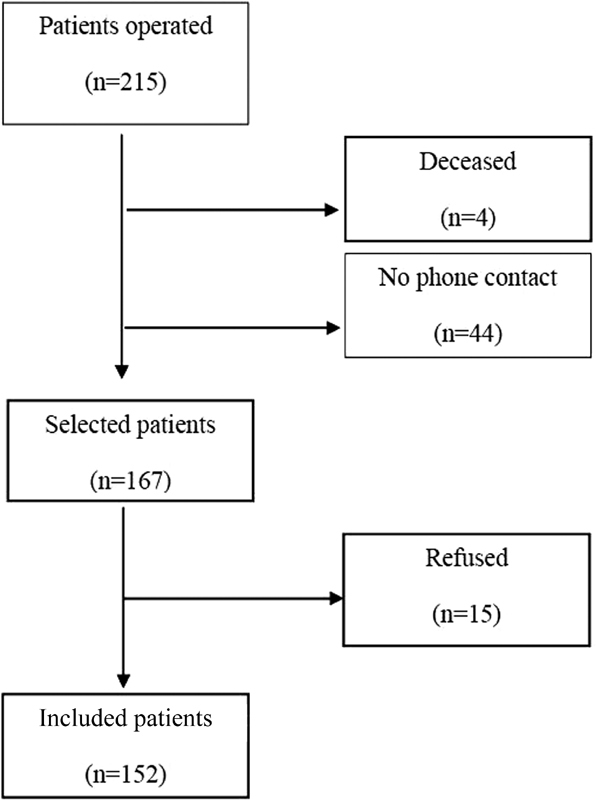
Flowchart of the selection of patients for the study.

A total of 215 patients were operated on by the same surgeon between January 2002 and December 2011 using the transobturator sling technique. Of these, 152 patients consented to participate in the study (Fig. 1). The mean follow-up time was 87 months (range: 60–127 months). The mean age was 51.8 years old (range: 27–85 years old). The mean body mass index (BMI) was 26.8 kg/m2 (range: 18.7–42.6 kg/m2) and the mean parity was 2.4 (0–10) (Table 1).

**Table 1:** Surgical data and complications

	n = 152	
Anesthesia	n	%
Spinal anesthesia	130	85.5
Epidural	21	13.8
General	1	0.7
**Bleeding**		
< 250 mL	142	98.6
> 250 mL	10	1.4
**Associated surgeries**		
Abdominal hysterectomy	6	3.9
Anterior colpoplasty	17	11.8
Posterior colpoplasty	62	40.7
**Complications**		
Foley > 24 hours	2	1.4
Local infection	5	3.4
Urinary tract infection	7	4.8
Mesh exposure	7	4.8
Groin pain	1	0.7
**Postoperative urgency**		
Yes	30	20.1
No	119	79.9
De novo urgency	14	9.2

Based on an ICIQ-SF score of zero, 99 (65.10%) patients were considered “cured”. Overall, 110 patients (73.02%) had ICIQ-SF scores ranging from 0 to 6 (the questionnaire has a maximum score of 21). Most patients (93.4%) would recommend the transobturator sling procedure, and only 10 patients (6.6%) would not recommend it.

The median value for valsalva leak point pressure (VLPP) was 90 cm H2O (range: 20–151 cm H2O). Of the patients who had surgical bleeding > 250 mL, only one required hemotransfusion due to posthysterectomy vaginal vault hematoma. The surgical data and complications are described in Table 1. The type of surgery most associated was posterior colpoplasty, and, in some patients, more than one type of surgery was performed. There were no cases of bladder or urethral injury. Two patients had postoperative voiding difficulty, and one had undergone urethrolysis. This patient had complete improvement of the symptoms and remained continent throughout the postoperative period. Only one patient had persistent groin pain, and, 60 days after surgery, had partial removal of the mesh. For this patient, the UI returned, but the pain ceased.

Of the 7 (4.6%) patients with mesh exposure, 6 had excision of the mesh under local anesthesia, and 1, under spinal anesthesia. A total of 5 patients developed infection at the surgical site, and were treated with oral antibiotics. One patient had dermalipectomy associated with the TOT sling and developed sepsis due to an abdominal wall infection; she was referred to the intensive care unit and received intravenous antibiotic therapy. The patients who complained of urgency in the postoperative period and did not have this symptom before the procedure were diagnosed with de novo urgency (14 patients, 9.2%). Of the 6 (3.9%) patients with previous surgery for UI (3 TOT, 1 TVT, and 2 Burch), only 2 had an ICIQ-SF score of 0.

In the analysis of the prognostic factors, only the variables of age and urgency were associated with a lower rate of subjective cure (ICIQ-SF score > 0). With each year of age, the chance of cure was 3% lower. The absence of postoperative urgency was associated with a six-fold increased likelihood of cure, in relation to the presence of urgency. Patients with previous surgery (6 patients - 3.9%) or surgery concurrent with the TOT sling procedure did not show a lower cure rate than those who had undergone only the TOT sling procedure. Likewise, increased BMI or the presence of VLPP < 60 mL did not affect the cure rate (Table 2).

**Table 2:** Univariate and multivariate analyses of cure prognostic factors (ICIQ-SF score = 0)

Prognostic factor	OR (univariate)	95%CI	*p*-value*	Adjusted OR (multivariate)	95%CI	Adjusted *p*-value
Clinical SUI	1.824	0.910–3.656	0.090			
Urodynamic SUI	3.404	0,.80–14.854	0.103			
Monarc versus Unitape	1.143	0.567–2.305	0.709			
BMI (kg/m^2^)	0.979	0.902–1.063	0.618			
Simultaneous surgery	1.001	0.502–1.994	0.998			
VLPP < 60 mL	1.240	0.518–2.971	0.629	0.374	0.115–1.221	0.103
Age (one year)	1.029	1.002–1.057	0.038	1.009	0.971–1.048	0.650
Previous surgery	2.341	0.954–5.748	0.063	2.592	0.791–8.495	0.116
Postoperative urgency	6.624	2.743–15.996	< 0.001	8.101	2.721–24.119	< 0.001

Abbreviations: 95%CI, 95% confidence interval; BMI, body mass index; ICIQ-SF, International Consultation on Incontinence Questionnaire-Short Form; OR, odds ratio; SUI, stress urinary incontinence; VLPP, valsalva leak point pressure.

Notes: *Wald test; *p* < 0.05 considered significant; multivariate enter model with input *p* = 0.20, including the constant; adjusted R^2^ of the multivariate model: 22%, Hosmer-Lemeshow test, *p* = 0.358.

**Fig. 2:**
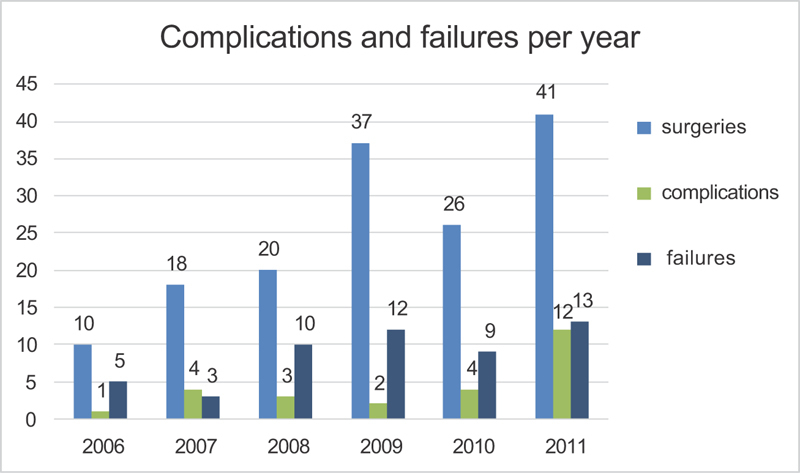
Complications and failures per year.

The graphical analysis of the rates of complications did not show an improvement in the cure rate or a decrease in complications with increased surgical experience (Fig. 2). The subjective cure rate, considering ICQ-SF = 0, was 62% in the first 36 months, and in the remaining 36 months, it was 64%, with no statistical difference. The complications that were statistically analyzed were surgical bleeding > 250 mL, groin pain, mesh exposure, surgical site infection, urinary tract infection, and voiding difficulty.

## Discussion

Knowledge of the long-term results of the TOT sling is essential to inform patients regarding the efficacy and safety of this surgery, which is one of the most frequently performed surgeries in the world for the treatment of SUI. The present study showed that the subjective cure rate for the TOT sling procedure in patients with SUI and MUI was 65.1% (using an ICIQ-SF score of 0 as the cure criterion), with an average follow-up time of 87 months. If an ICIQ-SF score of 0 to 6 was used as the cure criterion, the rate was 73%.^10^ However, in a set of separate questions, 94% of the patients described themselves as cured or improved, and 93% would recommend the surgery.

Our results are similar to those of other authors who evaluated the TOT sling after long-term follow-up.^11^ ^12^ However, in our study, we did not exclude patients with surgeries concomitant with TOT and reoperations. Studies with subjective cure rates higher than 90% usually assess patients with SUI alone and without concomitant prolapse.^13^ It is possible that this exclusion explains the higher cure rates.

We have observed that, with each year of the patient's life, there was a reduction of 3% in the subjective cure rate for the TOT sling procedure. Other authors have reported similar TOT sling cure rates in patients > 70 years old and in younger patients, but the presence of MUI changes this rate in older patients.^14^ ^15^ Aging is also associated with the emergence of other health problems that contribute to a less satisfactory outcome.^16^ It is not known if de novo urgency is due to the procedure or to a higher prevalence of detrusor hyperactivity with advancing age.^13^ ^17^ The emergence of de novo urgency after the TOT sling procedure affects patient satisfaction.^11^ ^12^ ^18^ The occurrence of de novo urgency in the present study was the most frequent complication (9.2%), and was correlated with decreased satisfaction with the surgery. Yonguc et al^19^ showed that the satisfaction rate for patients decreases within 5 years after the TOT sling procedure, and that the main reason for this is urge UI.

In patients with urodynamic SUI, the subjective cure rate after the TOT sling procedure was 5 times higher, and this finding is similar to that observed in a long-term study evaluating only TVT.^20^ The urgency increases over the years, from 17.3% 2 years postoperatively to 41.7% 10 years postoperatively. Therefore, patients with MUI should be informed, before the surgery, of the decreased chance of cure, and that urgency may develop with time.^20^

Among the complications, mesh exposure occurred in 4.8% of the patients, a finding similar to that reported by other authors.^11^ ^12^ ^18^ There were no cases of urethral or bladder injury, and the occurrence of groin pain (0.7%) was lower than that reported by other authors; this may be due to the surgeon's increased experience.^21^

The strong points of the present study are the long follow-up for the TOT surgery, the surgery performed by a single experienced surgeon, and the determination of the impact of the surgeon's experience on the results. In addition, the studied population represents the patients with UI who present themselves in outpatient clinics. The main limitation of our study is its retrospective character and the fact that the ICIQ-SF was not applied preoperatively in order for us to make a comparison with the postoperative results.

## Conclusion

The TOT sling procedure is an effective surgical treatment for UI, has a low rate of complications, and a high rate of patient satisfaction in the long term. The main risk factor for failure was de novo onset of urgency in the postoperative period. The increase in the surgeon's experience over time had no impact on the frequency of complications or on the improvement of the subjective cure rate. These results for a single surgeon are likely representative of the clinical practice.
